# Moiré-Enabled Topological Superconductivity

**DOI:** 10.1021/acs.nanolett.1c03856

**Published:** 2022-01-03

**Authors:** Shawulienu Kezilebieke, Viliam Vaňo, Md N. Huda, Markus Aapro, Somesh C. Ganguli, Peter Liljeroth, Jose L. Lado

**Affiliations:** † Department of Applied Physics, Aalto University, 00076 Aalto, Finland; ‡ Department of Physics, Department of Chemistry, and Nanoscience Center, University of Jyväskylä, FI-40014 Jyväskylä, Finland

**Keywords:** scanning tunneling microscopy, topological superconductor, moiré pattern, 2D ferromagnet

## Abstract

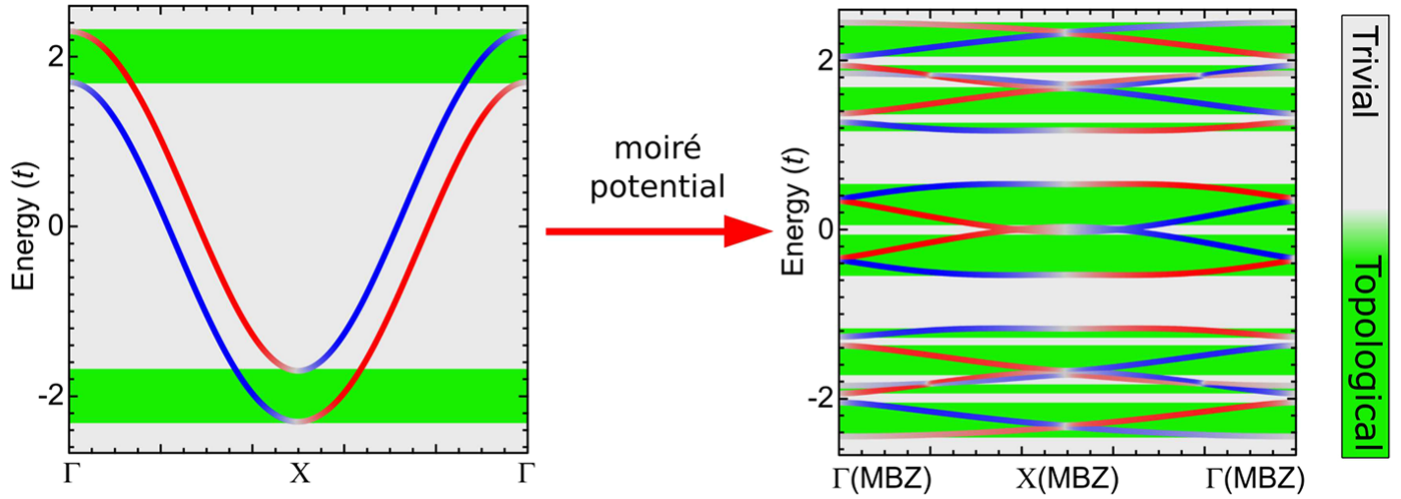

The search for artificial topological superconductivity has been limited by the stringent conditions required for its emergence. As exemplified by the recent discoveries of various correlated electronic states in twisted van der Waals materials, moiré patterns can act as a powerful knob to create artificial electronic structures. Here, we demonstrate that a moiré pattern between a van der Waals superconductor and a monolayer ferromagnet creates a periodic potential modulation that enables the realization of a topological superconducting state that would not be accessible in the absence of the moiré. The magnetic moiré pattern gives rise to Yu–Shiba–Rusinov minibands and periodic modulation of the Majorana edge modes that we detect using low-temperature scanning tunneling microscopy (STM) and spectroscopy (STS). Moiré patterns and, more broadly, periodic potential modulations are powerful tools to overcome the conventional constraints for realizing and controlling topological superconductivity.

There are many routes to realizing topological superconductivity in artificial structures,^1−10^ and perhaps the most widely used path uses the combination of superconductivity, spin–orbit coupling, and magnetism.^11,12^ This recipe has been recently used in a CrBr_3_/NbSe_2_ van der Waals (vdW) heterostructure, where the emergence of topological superconductivity was demonstrated.^13^ In stark contrast with the realization based on semiconducting nanowires,^14−16^ the electronic structure of the CrBr_3_/NbSe_2_ system features a highly doped band of NbSe_2_, far from the typical allowed regimes for topological superconductivity to appear. This system has a complex electronic structure combining a Fermi surface reconstruction of NbSe_2_ stemming from its charge density wave,^17^ together with a strong moiré arising from the lattice mismatch between NbSe_2_ and CrBr_3_. It is surprising that such complex electronic structure with no external control parameters turns out to give rise to a state featuring topological superconductivity. The emergence of topological superconductivity in a NbSe_2_/CrBr_3_ heterostructure^13^ can be rationalized as follows. The ferromagnetic state of CrBr_3_ induces an exchange field on the electronic structure of NbSe_2_, and the mirror symmetry breaking of the heterostructure creates a strong Rashba spin–orbit coupling in the NbSe_2_ bands. When including the intrinsic s-wave superconducting order of NbSe_2_, the low energy electronic structure harvests the three fundamental ingredients for the emergence of artificial topological superconductivity:^1−10^ s-wave superconducting order, Rashba spin–orbit coupling, and exchange fields. Such low-energy effective model has been shown to faithfully capture the phenomenology observed experimentally.^13^ However, interesting additional microscopic contributions have been so far unaddressed. First, Majorana edge modes showed strong regular modulation at the edges of the topological superconducting island. Second, the mismatch between the NbSe_2_ and CrBr_3_ monolayers gives rise to a moiré pattern modulating all the parameters in space. And finally, the emergence of topological superconductivity in the minimal model required a delicate fine-tuning of the NbSe_2_ Fermi level. Here we extend our earlier experimental results on the CrBr_3_/NbSe_2_ system, demonstrating how the previous three features are naturally accounted by emergent moiré phenomena of the heterostructure.

Here, we show that the apparent complexity created by the moiré pattern in the CrBr_3_/NbSe_2_ system can be the ultimate driving force of its topological superconducting state. In particular, the strongly modulated electrostatic potential and exchange coupling in the moiré heterostructure give rise to modulated Yu–Shiba–Rusinov (YSR) bands that allow for the emergence of topological superconductivity in generic regimes where it is otherwise forbidden. We explain theoretically and demonstrate experimentally that the moiré modulation of the topological state emerging from the YSR bands is also visible in the spatial distribution of the one-dimensional topological Majorana modes. Our results put moiré physics forward as a powerful knob enabling topological superconductivity. Finally, conceptually similar effects can be realized by creating a periodic potential modulation (e.g., through external gating) in semiconducting devices,^10,14,15,18−25^ which offers new ways of controlling topological superconductivity toward the realization of topological qubits in the future.

To understand the potential of moiré modulations for driving topological superconductivity, we consider a generic model incorporating long-wavelength modulations in its different parameters.^26^ Specifically, we take a Hamiltonian that includes all the known ingredients for topological superconductivity: s-wave superconductivity, Rashba spin–orbit coupling, and ferromagnetism.^1−3,12,27^ We introduce the moiré modulation through spatial variation of the parameters of the tight-binding model: on-site energies, the hoppings, the exchange coupling, Rashba spin–orbit coupling, and the s-wave superconductivity (details are given in the Supporting Information). Despite the increasing complexity of the Hamiltonian *H* from having spatially dependent order parameters, their effects on enabling a topological superconducting state in arbitrary conditions can be easily rationalized. In order to illustrate these possibilities, we first focus on a minimal case: a one-dimensional moiré system (Figure 1a).

**Figure 1:**
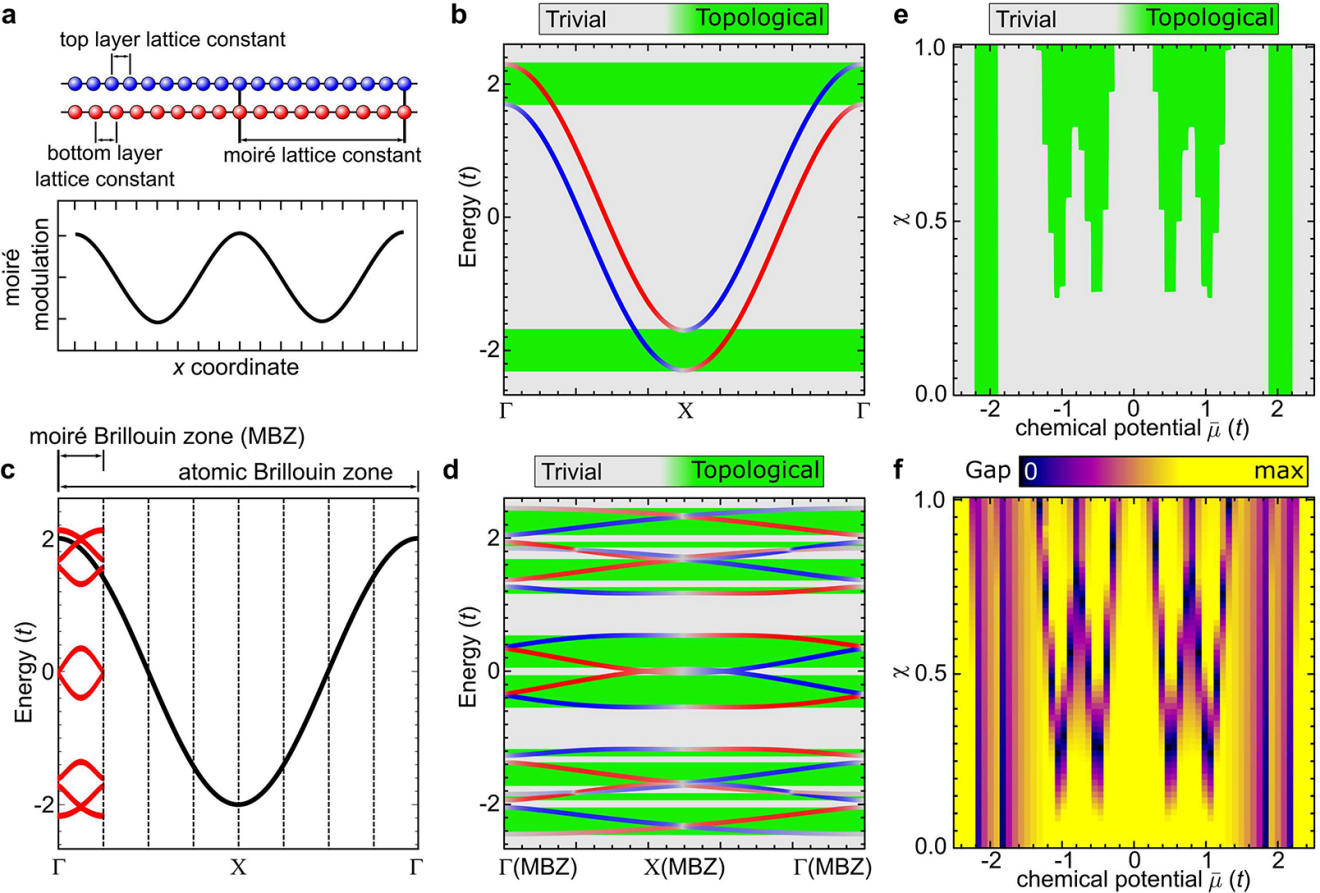
Moiré-driven topological superconductivity. (a) Schematic of the moiré pattern set up by the lattice mismatch, and the resulting modulation of the on-site energies, the exchange coupling, and other parameters depending on the registry between the two lattices. (b) Pseudohelical states appear in the presence of Rashba and exchange interactions at the top and bottom of the band of a one-dimensional model, giving rise to topological superconductivity (green). (c) Band structure of a simple one-dimensional model before (black line) and after (red lines) turning on the moiré modulation. (d) Including the effect of the moiré pattern enables pseudohelical states and hence topological superconductivity to emerge at the top and bottom of the moiré minibands. Here, the moiré modulation is given by a modulation of the on-site energies. (e, f) Topological phase diagram (e) and the gap (f) for a 1D chain as a function of the μ̅ and the modulation χ of exchange.

For a one-dimensional model with uniform order parameters, topological superconductivity can only appear at the top bottom of the band, as shown in Figure 1b. This is associated with a single set of pseudohelical states that develop at the top and bottom of the band in the presence of Rashba spin–orbit interaction and exchange coupling. Turning on a moiré modulation in the chemical potential μ(**r**) ∼ cos(Ω*x*), for a given wavevector Ω, will cause folding of the band structure and opening of minigaps between the folded bands as illustrated in Figure 1c.^26,28−30^ As shown in Figure 1c, there are additional band tops and bottoms, where topological superconductivity can potentially be realized. Indeed, when Rashba spin–orbit coupling, exchange, and superconductivity are included in addition to the moiré pattern, pseudohelical states appear close to charge neutrality (Figure 1d), allowing for the emergence of topological superconductivity. This leads to topological regions in the phase diagram (Figure 1e) at values of chemical potential corresponding to a topologically trivial state in the absence of the moiré modulation. Associated with new topological regions, gap closing and reopening are driven by the moiré modulation as shown in Figure 1f. It is important to emphasize that in the absence of the moiré modulation, no topological superconducting state can be created at all in this energy range. While this example uses a modulation of the chemical potential, modulation in either exchange, Rashba, hoppings, or proximity superconductivity is effective to drive these moiré-enabled topological phase transitions (Figure S1). This idea provides a new direction to explore topological superconductivity in designed one-dimensional systems, such as nanowires grown with a long-range modulation,^31,32^ in doping regimes in which it would not be allowed otherwise.

This phenomenology can be extended to two-dimensional systems that naturally arise due to the moiré modulation in van der Waals heterostructures. Figure 2a shows an atomically resolved STM image of the CrBr_3_ monolayer grown on a bulk NbSe_2_ substrate (see Supporting Information for experimental details), revealing a well-ordered moiré superstructure with 6.3 nm periodicity arising from the lattice mismatch between the CrBr_3_ and the NbSe_2_ layers.^33^ The moiré pattern matches a structure with 19 NbSe_2_ unit cells accommodating 10 unit cells of CrBr_3_, thus forming a 6.3 nm × 6.3 nm superstructure. This also matches the measured lattice constants of CrBr_3_ and NbSe_2_.

**Figure 2:**
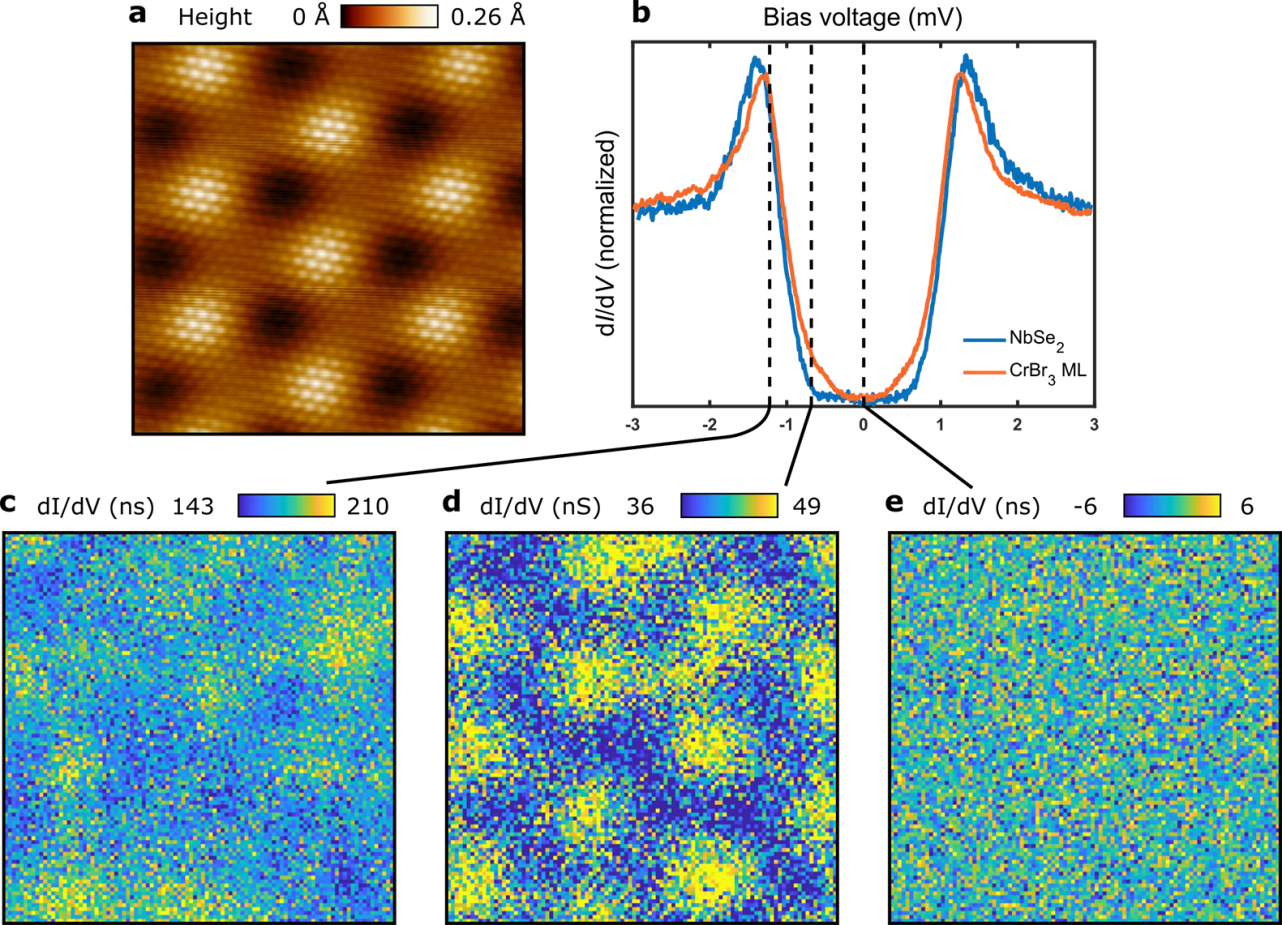
Correlation between moiré pattern and YSR bands. (a) STM image of CrBr_3_ ML on NbSe_2_ obtained at *V* = 1.5 V and *I* = 300 pA. Image size is 20 × 20 nm^2^. (b) d*I*/d*V* spectroscopy on the NbSe_2_ substrate (blue) and on the island of CrBr_3_ ML (orange). (c–e) d*I*/d*V* maps (from spectral grid) at different *V* values highlighted in (b). The maps correspond to the STM image in (a), and each map is plotted on a separate color scale. A clear correlation between the moiré pattern and d*I*/d*V* map only appears at the energy of the YSR bands (*V* = 0.8 mV). The grid map measurement conditions are the following: *V* = 3 mV, *I* = 300 pA, and *T* = 350 mK.

The interaction of the magnetism of the CrBr_3_ layer^13,33,34^ with the superconductivity from the NbSe_2_ substrate gives rise to the YSR bands inside the superconducting gap that are also modulated by the moiré pattern. The formation of YSR band is shown in Figure 2b (orange line) where the d*I*/d*V* spectrum taken in the middle of the CrBr_3_ island has a pair of conductance onsets at ±0.35 mV. This spectroscopic signature can be compared to a d*I*/d*V* spectrum of bare NbSe_2_, where a hard gap with an extended region of zero differential conductance around zero bias is observed (Figure 2b, blue line). By subtracting the background spectra from the two-band model fit, we obtain the experimental topological gap that is around Δ_t_ ≈ 0.3Δ (see Supporting Information). In order to visualize the spatial modulation of the YSR band, we have recorded grid d*I*/d*V* spectroscopy maps (Figure 2c–e) over the area shown in the Figure 2a. The d*I*/d*V* maps exhibit periodic modulation of the signal intensity over the moiré unit cell only at the energy of the YSR bands. This is caused by the intensity variations of the YSR band local density of states (LDOS) rather than energy variations of the YSR band as further demonstrated in the Supporting Information (Figures S7 and S8).

The microscopic origin of the variations of the YSR band intensities can be easily rationalized. First, the modulation of exchange (Figure 3a) stems from the strong dependence of superexchange interactions on the local stacking, as demonstrated in CrI_3_ and CrBr_3_ bilayers.^32,35,36^ This feature suggests that the moiré pattern not only modulates the absolute value of the effective exchange but also can change its sign.^35,36^ Second, as a consequence of the modulation of the exchange field, the superconducting order parameter will also be modulated in the opposite way, due to the competition of s-wave superconductivity from NbSe_2_ and the proximity induced exchange field.^37^ Third, the modulation of the onsite energies stems from an electrostatic effect associated with the stacking. We can directly measure the modulation of this electrostatic potential through the spatial modulation of the conduction band edge of CrBr_3_ (Figure S9). Fourth, modulations in the hoppings are expected from small relaxation effects, well-known in other dichalcogenide-based twisted systems.^38,39^ Fifth, the charge density wave of NbSe_2_^17^ will introduce additional short-range modulation in both the hopping and local onsite energies.^40,41^ While all these effects can be incorporated into the effective model, we can reproduce the experimental results even using a minimal model that only incorporates spatially varying exchange interactions and onsite energies (Figure 3a). This is supported by the fact that a simple triangular lattice nearest neighbor tight binding model gives a good representation of the Fermi surface of NbSe_2_ in the presence of Ising and Rashba SOCs and the CDW reconstruction (see Supporting Information section “Realistic tight-binding model for CrBr_3_/NbSe_2_ heterostructure” for details). This results in a topological superconducting band structure (Figure 3b) in a chemical potential range close to charge neutrality, where the system is trivial in the absence of moiré modulations. Associated with these modulations, moiré-modulated YSR bands emerge (Figure 3c). The theory predicts (in agreement with our experimental results) that the moiré pattern gives rise to a spatial modulation of the intensity (Figure 3d) of the in-gap states but not of their energies.

**Figure 3:**
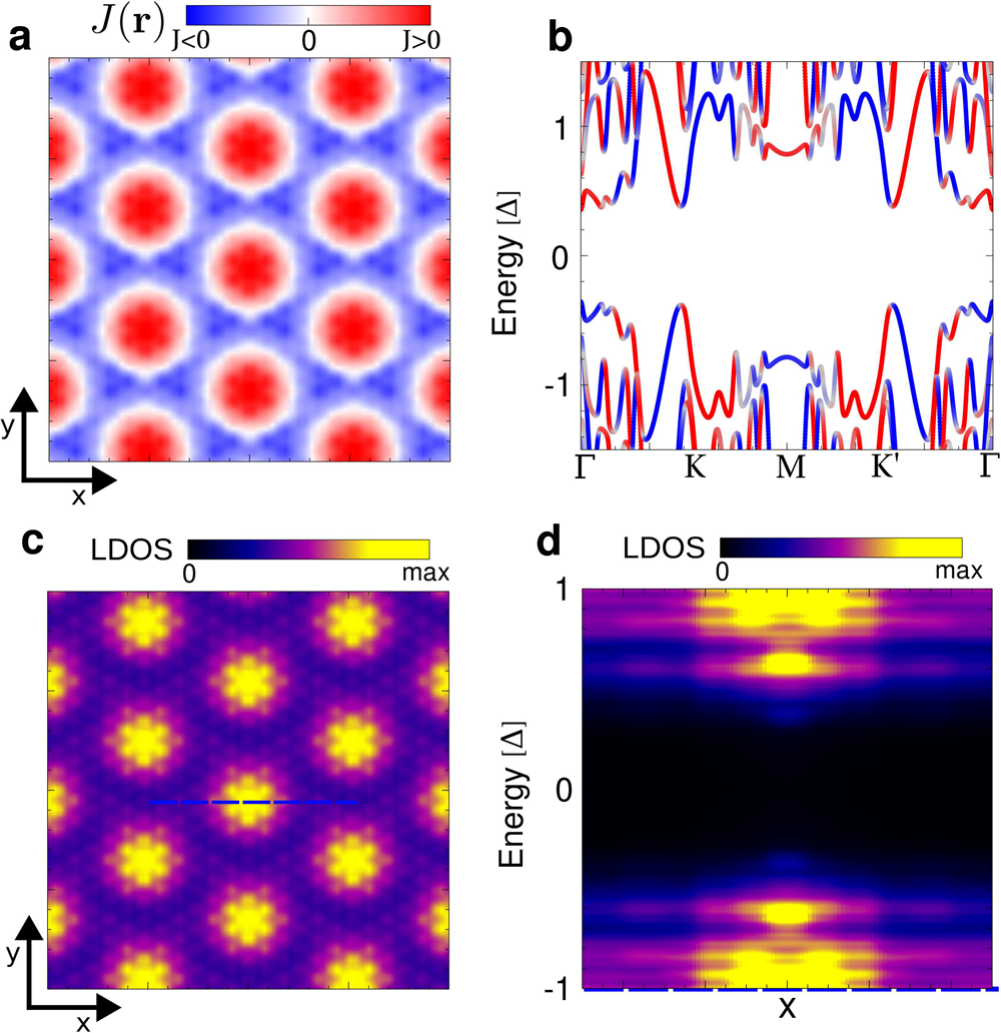
Moiré-enabled topological superconductivity: mechanism in 2D topological superconductor NbSe_2_/CrBr_3_. (a) Moiré profile for the exchange coupling *J*(**r**). (b) Resulting band structure in the topological regime with spatially varying exchange as described in the text. (c) Modulations of the YSR band LDOS due to the modulated exchange. (d) Spatial modulation of the bulk YSR bands along the line indicated in (c).

Above, we have focused on the impact of the moiré pattern on the bulk electronic structure, but the moiré electronic structure also gets imprinted on the topological edge modes. In particular, the emergence of a YSR moiré band structure suggests that topological edge modes may inherit the moiré distribution of the bulk YSR states. We now focus on the edge of a CrBr_3_ island, as shown in Figure 4a. At biases at and above the YSR bands (Figure 4b), no strong modulation at the edge is observed. In stark contrast, when taking energies inside the topological gap, we observe topological edge modes with a strong modulation with the period of the moiré pattern (Figure 4c). This is also visible in the single d*I*/d*V* spectra extracted at the points corresponding to the minimum and maximum intensity along the edge (points marked in Figure 4c, spectra shown in Figure 4d). We have analyzed a corresponding finite-size structure with our theoretical model (details in the Supporting Information) as shown in Figure 4e,f. As expected, at energies above the topological gap, modulated YSR states appear (Figure 4e). In strong correspondence with the experimental results, inside the gap, strongly modulated in-gap modes dominate the spectra (Figure 4f). This direct relationship between the edge modes and the bulk moiré modulation demonstrates a nontrivial role of the moiré pattern in creating the topological superconducting state. The moiré-induced topological phase transition and the modulation of edge modes are general features of the physical picture and will occur for the one-dimensional and two-dimensional realizations of these systems. This provides an experimentally simple way of verifying the presence and assessing the impact of the moiré modulation on the topological superconducting state.

**Figure 4:**
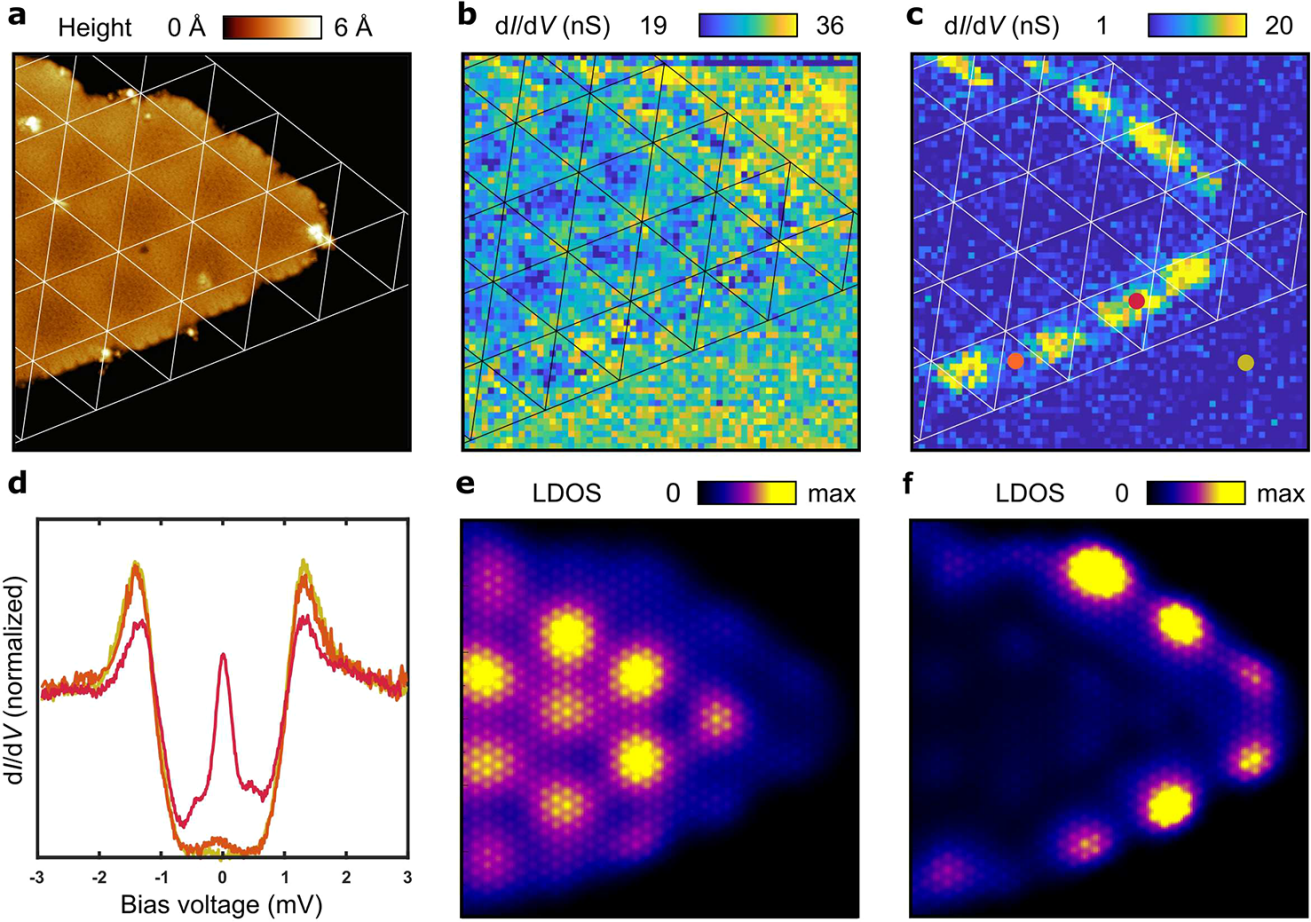
Edge states of the topological superconductor in van der Waals heterostructure with a moiré pattern. (a) 33 × 33 nm^2^ STM image of CrBr_3_ island on NbSe_2_, obtained at *V* = 1.0 V and *I* = 10 pA. (b) d*I*/d*V* map at energy of the YSR bands *V* = 0.8 mV with a moiré pattern appearing in the bulk of CrBr_3_ island. (c) d*I*/d*V* map (from spectral grid) at *V* = 0 mV showing Majorana edge modes. (d) d*I*/d*V* spectra acquired at the positions indicated in (c) (red, strong edge mode intensity; orange, weak edge mode intensity; yellow, background spectrum on NbSe_2_. (e, f) Theorically computed LDOS in the presence of a moiré exchange, at the energies of the YSR states (e) and the Majorana zero modes (f) for an island that closely mimics the shape of the studied experimentally. The grid map measurement conditions are the following: *V* = 3 mV, *I* = 140 pA, and *T* = 350 mK.

To summarize, we have demonstrated that moiré modulations allow realization of topological superconductivity in parameter regimes otherwise forbidden by the electronic structure. In particular, by accounting for the moire modulation, we have solved three open questions on the emergence of topological superconductivity in CrBr_3_/NbSe_2_. First, the spatial modulation of the edge modes directly corresponds to the moiré modulation of the bulk Yu–Shiba–Rusinov bands. Second, there is no need for the fine-tuning of the chemical potential as the moiré modulation results in a topological phase around charge neutrality over a broad range of the values of the chemical potential. And third, the detrimental effect of the Ising spin–orbit interaction is mostly removed by the modification of the band structure due to the charge-density wave modulation of the NbSe_2_. Concomitant to this moiré-enabled superconducting state, moiré-modulated YSR bands appear, whose topological band structure is ultimately responsible for the topological superconducting state. We have demonstrated this idea in a CrBr_3_/NbSe_2_ twisted heterostructure, showing the emergence of moiré YSR bands and moiré-modulated edge modes, the two paradigmatic experimental signatures of a moiré-enabled topological state. Moiré-enabled topological phase transitions are especially powerful in twisted van der Waals heterostructures, where the twist angle can be used as a knob to push the system to a topological superconducting state. Our results demonstrate the possibility of using twist engineering to design topological quantum materials with a high potential for creating a platform for realizing strongly interacting topological superconductors. This provides a new paradigmatic direction in the field of topological twistronics.

## Supporting Information Available

Experimental methods and additional experimental and theoretical results (PDF)
