# Supplementary material for: Moiré-Enabled Topological Superconductivity
Source: Nano Lett. 2022 Jan 3;22(1):328–33. doi: 10.1021/acs.nanolett.1c03856 (PMC8759081; doi:10.1021/acs.nanolett.1c03856)
Supplement: Supplementary file 1 — nl1c03856_si_001.pdf [file nl1c03856_si_001.pdf]

Supporting Information: Moiré-enabled topological superconductivity

Shawulienu Kezilebieke,*,†,‡ Viliam Vaňo,† Md N. Huda,† Markus Aapro,† Somesh C. Ganguli,† Peter Liljeroth,† and Jose L. Lado*,†

†*Department of Applied Physics, Aalto University, 00076 Aalto, Finland*

‡*Department of Physics, Department of Chemistry and Nanoscience Center, University of Jyväskylä, FI-40014 University of Jyväskylä, Finland*

E-mail: kezilebieke.a.shawulienu@jyu.fi; jose.lado@aalto.fi

Methods

Scanning tunneling microscopy (STM) and spectroscopy (STS) measurements: After the sample preparation, it was inserted into a low-temperature STM (Unisoku USM-1300) housed in the same UHV system and all subsequent experiments were performed at $T = 350$ mK. STM images were taken in constant-current mode. $\mathrm{d}I/\mathrm{d}V_\mathrm{b}$ spectra were recorded by standard lock-in detection while sweeping the sample bias in an open feedback loop configuration, with a peak-to-peak bias modulation of 30-50 μV for a small bias range and 10 mV for a larger bias range, respectively, at a frequency of 707 Hz. Spectra from grid spectroscopy experiments were normalized by the normal state conductance, i.e. $\mathrm{d}I/\mathrm{d}V_\mathrm{b}$ at a bias voltage corresponding to a few times the superconducting gap.

Sample preparation: The details of the sample preparation can be found in.[S1] Briefly, the $CrBr_3$ thin film was grown on a freshly cleaved $NbSe_2$ substrate by compound source molecular beam epitaxy (MBE) by evaporating anhydrous $CrBr_3$ powder from a Knudsen

cell. The growth speed was determined by checking the coverage of the as-grown samples by scanning tunneling microscopy (STM). The optimal substrate temperature for the growth of $CrBr_3$ monolayer films was $\sim 270°C$.

Theoretical model

We model the moiré modulated system by a tight binding (TB) model in a triangular lattice of the form

$$\begin{aligned} H = \sum_{\langle ij \rangle, s} t\left(\frac{\mathbf{r}_i + \mathbf{r}_j}{2}\right) c^\dagger_{i,s} c_{j,s} + \sum_{i,s} \mu(\mathbf{r}) c^\dagger_{i,s} c_{i,s} + \\ i \sum_{\langle ij \rangle, s, s'} \lambda \left(\frac{\mathbf{r}_i + \mathbf{r}_j}{2}\right) \mathbf{d}_{ij} \cdot \sigma^{s,s'} c^\dagger_{i,s} c_{j,s'} + \\ \sum_{i,s,s'} J(\mathbf{r}_i) \sigma_z^{s,s'} c^\dagger_{i,s} c_{i,s'} + \sum_i \Delta(\mathbf{r}_i) c^\dagger_{i,\uparrow} c^\dagger_{i,\downarrow} + \text{h.c.} \end{aligned} \tag{1}$$

where $\langle\rangle$ denotes sum over first neighbors, $c^\dagger_{i,s}$ is the creation operator of an electron with spin s in site i, $\mathbf{d}_{ij} = \mathbf{r}_i - \mathbf{r}_j$, $\mu(\mathbf{r})$ is the spatially dependent band off-set, $J(\mathbf{r})$ is the spatially dependent exchange coupling, and $\Delta(\mathbf{r})$ is the spatially dependent superconducting order. We denote the different average value in the moiré unit cell as

$$\begin{aligned} \bar{t} &= \langle t(\mathbf{r}_i) \rangle_{\mathbf{r}_i} \\ \bar{J} &= \langle J(\mathbf{r}_i) \rangle_{\mathbf{r}_i} \\ \bar{\Delta} &= \langle \Delta(\mathbf{r}_i) \rangle_{\mathbf{r}_i} \\ \bar{\lambda} &= \langle \lambda(\mathbf{r}_i) \rangle_{\mathbf{r}_i} \\ \bar{\mu} &= \langle \mu(\mathbf{r}_i) \rangle_{\mathbf{r}_i} \end{aligned} \tag{2}$$

and their spatial variation as

$$\begin{aligned}\delta t &= \max(t(\mathbf{r}_i)) - \min(t(\mathbf{r}_i)) \\ \delta J &= \max(J(\mathbf{r}_i)) - \min(J(\mathbf{r}_i)) \\ \delta \Delta &= \max(\Delta(\mathbf{r}_i)) - \min(\Delta(\mathbf{r}_i)) \\ \delta \lambda &= \max(\lambda(\mathbf{r}_i)) - \min(\lambda(\mathbf{r}_i)) \\ \delta \mu &= \max(\mu(\mathbf{r}_i)) - \min(\mu(\mathbf{r}_i))\end{aligned} \tag{3}$$

If the physics created by the modulation is ignored, all the different phases would depend on the average values $\bar{X}$, with no dependence on the spatial variations $\chi \equiv \delta X$.

For the two-dimensional model used to capture the moiré physics, we include the modulation of the different terms in the Hamiltonian by taking a spatial profile that respects the C_3 symmetry of the structure

$$\Xi(\mathbf{r}) = c_0 + c_1 \sum_{n=1}^{3} \cos\left(R^n \mathbf{k} \cdot \mathbf{r} + \phi\right) \tag{4}$$

where R is the 120° rotation matrix, k is the wavevector of the modulation and ϕ a shift in the pattern. By definition, the previous functional form displays the C_3 symmetry of the structure, and for $\phi = 0$ the symmetry becomes C_6. The previous functional form can thus be used to parametrize different terms of the Hamiltonian, including exchange, Rashba, onsite modulations, superconducting proximity effect or strain.

Moiré-induced topological phase transition in 1D

It is instructive to first focus on how a topological phase transition can be induced in a one-dimensional system just by switching on the moiré potential. For the sake of that, we will take the Hamiltonian of Eq. 1 for a linear tight-binding chain, and explore how the system develops a topological phase as a moiré potential in different terms is switched on. We will take a modulation of the form $\Xi(\mathbf{r}) = c_0 + c_1 \cos \mathbf{k} \cdot \mathbf{r}$ and we will focus on three specific

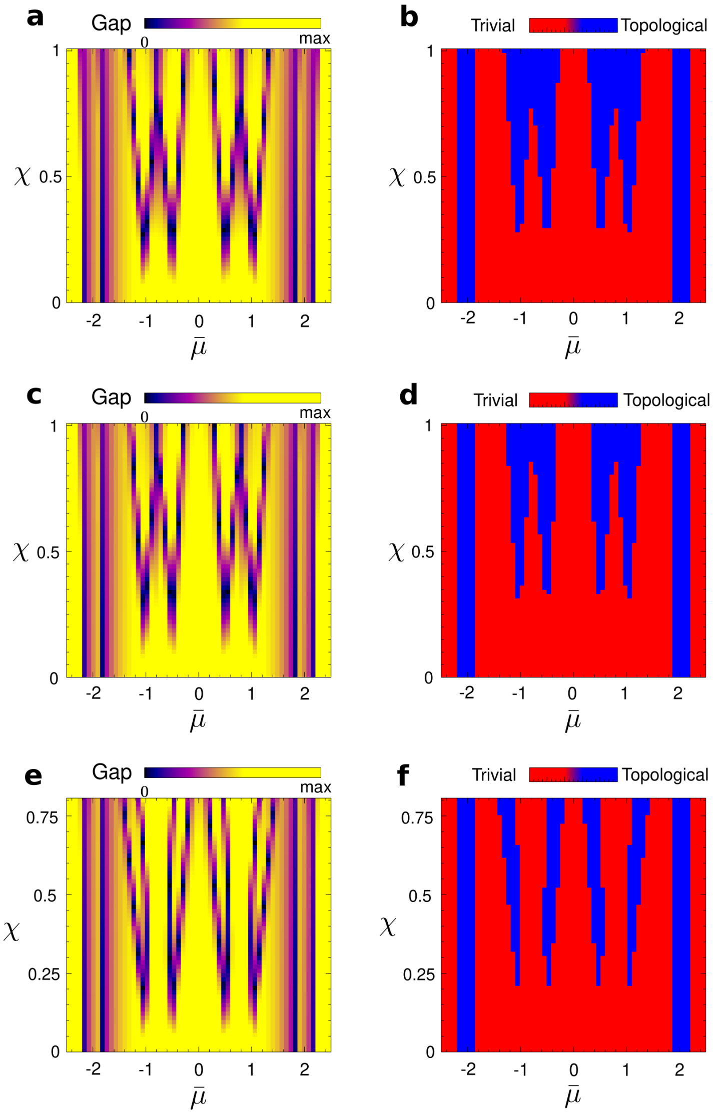

Figure S1: Gap (a,c,e) and topological phase diagram (b,d,f) for a 1D chain as function of the $\bar{\mu}$ and the the modulation χ of exchange (a,b), exchange and superconductivity (c,d) and hopping (e,f). For each case, the rest of the parameters are kept unmodulated. It is observed that as the modulation strength χ is increased, new topological regions appear in the phase diagram (b,d,f), that are acompanied by gap closings (a,c,e). We took $\delta J = \chi$ for (a,b), $\delta J = \chi, \delta\Delta = \chi/2$ for (c,d) and $\delta t = \chi$ for (e,f).

cases (Fig. S1): i) modulation in the exchange (Fig. S1a,b), ii) modulation in the exchange and superconducting proximity (Fig. S1c,d), iii) modulation in the hopping (Fig. S1e,f). For each one of the previous cases, the rest of the parameters $\bar{\lambda}, \bar{J}, \bar{\Delta}, \bar{t}$ are kept constant and without modulation. We will explore the topological phase diagram as a function of the chemical potential $\bar{\mu}$ of the chain, which highlights the non-trivial impact of the modulation

at the different fillings. In the absence of any modulation, the previous model displays a topological superconducting state only at the top and bottom of the band. As the modulation in any of the parameters is switched on, a new topological regions appear, demonstrating that any of those modulations is capable of driving a topological superconducting state.

Moiré-induced topological phase transition in a simple triangular lattice model

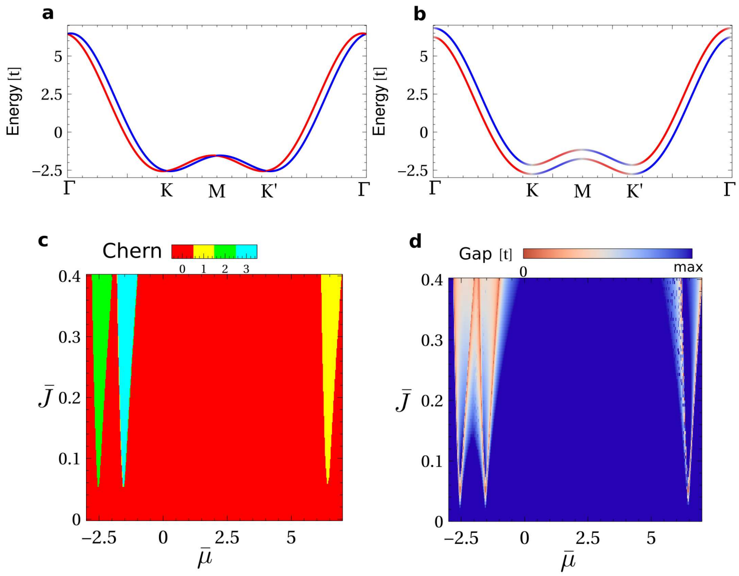

Figure S2: Band structure for a triangular lattice in a NN TB model with Rashba (a), and with Rashba and exchange field (b), where it is shown that pseudo helical states appear at Γ, M and K. Panel (c) shows the topological phase diagram, showing that at the energies of those points a topological states appears, which is accompanied by a gap closing and reopening (d). It is clearly observed that close to charge neutrality no topological state appears.

Unmodulated limit

We now move on to consider how the moiré modulation can drive a topological phase transition in a two-dimensional model using a nearest neighbor (NN) tight-binding model on a triangular lattice. As a starting point, it is instructive to consider the phase diagram for a simple triangular lattice model without modulation, shown in Fig. S2.

This would correspond to taking $\delta\mu = \delta\lambda = \delta J = \delta t = \delta\Delta = 0$. The topological regions appear when the chemical potential crosses a parabolic band that happens at the bottom of the spectra (K point), below charge neutrality (M points) and at the top of the band (Γ point). This can be understood from the fact that at those high symmetry points, the combination of Rashba spin-orbit coupling and exchange field creates pseudo-helical states, that can be then gapped in a topologically non-trivial way when s-wave superconductivity is turned on.[S2] In particular, it is seen that around charge neutrality (above the M point maxima), no topological state appears by increasing the exchange coupling.

Modulated exchange

We now move on to the case in which a modulation in the model is present , and we focus on the region around charge neutrality, which was before shown to be topologically trivial (Fig. S2). We focus first on a minimal case, in which the only modulated term is the exchange, with all the other terms uniform in space. We take the exchange profile of Fig. S3a, with $\bar{\lambda} = 4\bar{\Delta}$, with an average value of $\bar{J} = 1.25\Delta$ and a modulation $\delta J = 4\bar{\lambda}$, leading to a profile positive at the center of the supercell, and with an opposite sign in the hexagonal-like spots. We take a 6×6 supercell for the tight binding model, the chemical potential at half filling of the supercell. In the absence of exchange field, the electronic structure shows a topologically trivial gap, as shown in Fig. S3b. If the non-uniform exchange is switched on, taking zero Rashba spin-orbit coupling, the electronic structure shows gapless Yu-Shiba-Rusinov states (Fig. S3c). When the the Rashba spin-orbit coupling is included, a gap opens up in the YSR bands, leading to the emergence of a topological gap with Chern number $\mathcal{C} = 2$ at half filling of the triangular superlattice (Fig. S3c). The topological gap obtained is of magnitude 0.2Δ. It must be emphasized that taking $\delta J = 0$ in the previous case would lead to a topologically trivial state. The previous results demonstrate that the existence of a modulated exchange alone allows to create a topological superconducting state in an otherwise forbidden region, even with the rest of the parameters constant.

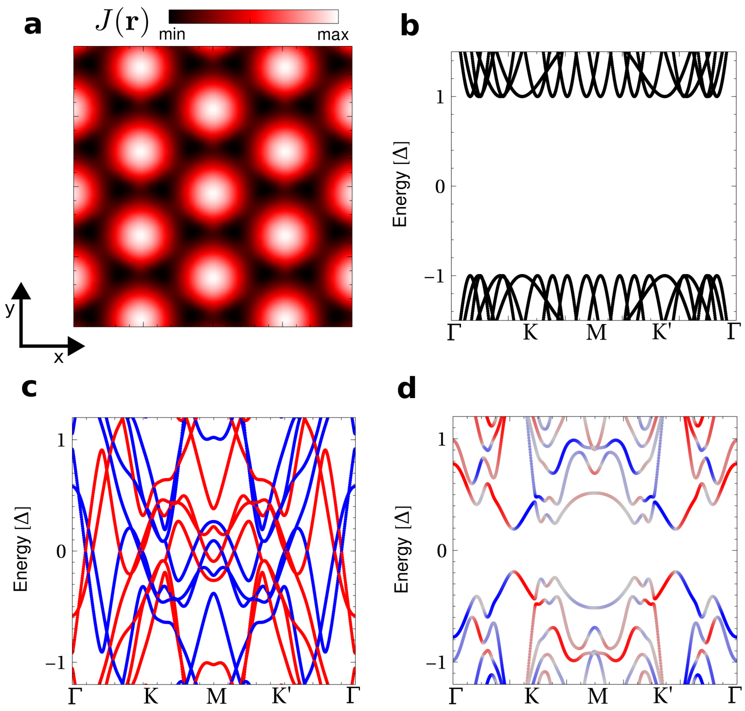

Figure S3: (a) Spatial profile of the exchange for a supercell, the rest of the parameters of the Hamiltonian will be taken constant and uniform in space. Panel (b) shows the band of the supercell in the absence of exchange, showing a topological trivial gap form the s-wave superconducting order. Panel (c) shows the bandstructure is the absence of Rashba SOC, showing gapless YSR states. Panel (d) shows the bandstructure with all the terms included, showing the emergence of a topological gap.

Modulated exchange and potential

In the following, we will show that the previous phenomenology also emerges when the exchange and local potential is modulated. This is the case shown in the main manuscript, associated with a topological state with Chern number $\mathcal{C} = 3$. In particular, we now explore the phase diagram in which the chemical potential μ and the exchange J are modulated. We will keep all the average values constant, and we will increase both δJ and $\delta\mu$ with a control parameter χ as $\delta J \sim \chi$ and $\delta\mu \sim \chi$. The phase diagram of the triangular lattice around charge neutrality is shown in Fig. S4a. It is clearly seen that as the modulation strength is increased, a topological region emerges at charge neutrality. A gap closing appears associated with the emergence of this topological region as shown in Fig. S4b. Let us now focus on two specific points of the phase diagram, one trivial (Fig. S4c,e) and one topological (Fig. S4d,f).

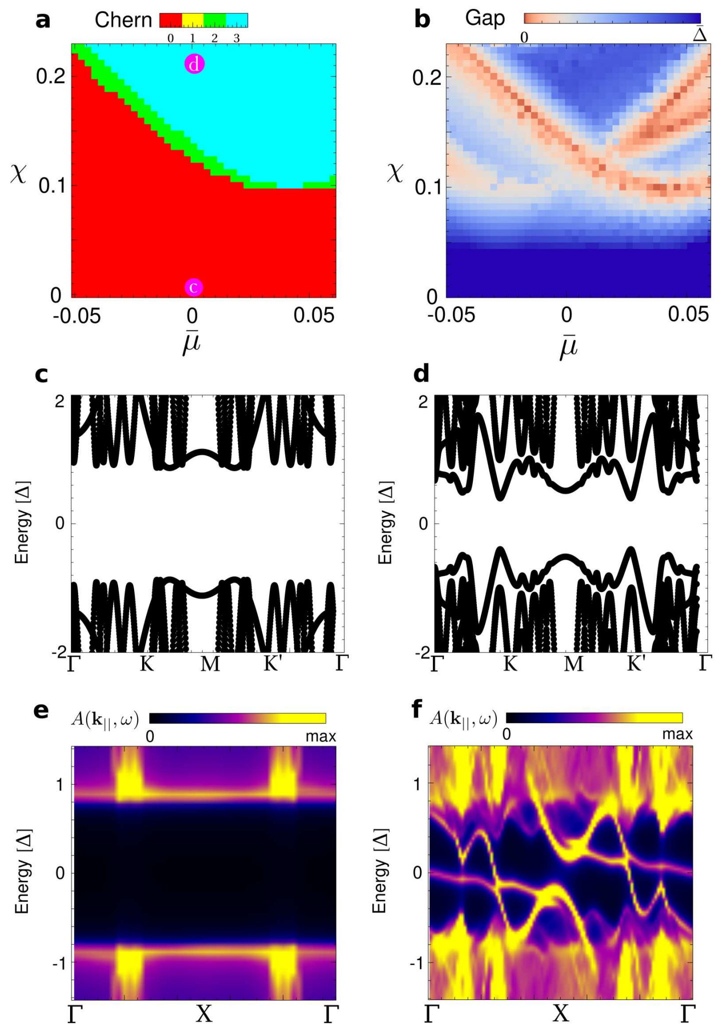

Figure S4: Topological phase diagram (a) and gap (b) for the NN TB triangular 2D lattice model, as a function of the average chemical potential $\bar{\mu}$ and the modulation strength χ. The modulation of the exchange and onsite energies are given by $\delta J = \chi$ and $\delta\mu = 4\chi$, and the other parameters are kept unmodulated and constant $\bar{J} = 0.2t$, $\bar{\Delta} = 0.05t$ and $\bar{\lambda} = 0.3t$. It is clearly observed that as the modulation is switched on, a topological states appears. Panels (c,d) show the band structures for a trivial (c) and topological (d) points in the phase diagram. The surface spectral functions are shown in (e,f), that show the absence of surface modes for the trivial case (e) and their presence for (f).

The band structures of the supercells are shown in Fig. S4c,d, that both feature a set of minibands. Whereas distinguishing between the two cases is not simple from their band structure, a dramatically different spectra would be observed at the edges. In particular, in Fig. S4e,f we show the spectral functions $A(\mathbf{r}, \omega)$ for a semi-infinite slab for the previous two cases, showing that the trivial case does not show in-gap edge modes (Fig. S4e), whereas the

topological one features topological Majorana modes (Fig. S4f).

We finally comment on the real-space profile of the edge states. The moiré pattern effectively creates a new lattice, whose lattice constant is determined by the moiré length scale, leading to a electronic structure with a periodicity of the moiré length. In particular, this implies that both the YSR states, and their associated edge modes will be modulated following the moire length. The dispersion of the edge mode now is associated with the quasi-momentum of the moiré lattice, in contrast with the unmodulated case in which it is associated with the quasi-momentum of the original atomic lattice. We note that this scenario is analogous to the Chern insulating state observed in twisted graphene bilayers, in which the edge states emerge associated with the new moiré length scale.[S3] Of course, directly probing the dispersion of the edge mode via quasi-particle interference would be challenging for this system. Technically, the emergence of chiral modes can be probed via thermal conductivity measurements in chiral topological superconductors.[S4] While this would be certainly interesting, such measurements are extremely challenging and well beyond the scope of our work. We finally want to note that our approach for addressing the emergence of topological modes is analogous to those used in other STM studies of topological superconductors with chiral edge states,[S5] in which also demonstrating the chirality of the states remains an open challenge.

Moiré Yu-Shiba-Rusinov bands

In this section, we address the relationship between the modulated exchange coupling in the twisted structure and the emergence of Yu-Shiba-Rusinov moiré bands (Fig. S5). It is first worth recalling that the modulated stacking in the supercell is expected to be the ultimate driving force of the modulated exchange coupling. In particular, the stacking can change not only the effective exchange coupling but also its sign. Here, we will show how this effect easily accounts for the emergence of moiré Yu-Shiba-Rusinov bands using an effective model. Even though we here will focus on an effective model, it is worth to mention that

a quantitative prediction of the change of exchange couplings with the stacking could be performed by first-principles methods.

For the sake of concreteness we will focus on a profile for the exchange coupling with C_6 symmetry, as shown in Fig. S5a. The density of states (Fig. S5b) of such a modulated system is shown in Fig. S5b, which features both a superconducting gap and in-gap YSR bands. It is observed that a strong modulation of the in-gap YSR modes appear (Fig. S5c), reflecting the original modulation of the exchange coupling (Fig. S5a). In stark contrast, above the superconducting gap the modulation of the states is shown to be much weaker (Fig. S5d). This phenomenology can be rationalized from the fact the in-gap YSR modes will be strongly dependent on the exchange coupling, whereas states above the gap are much more insensitive to such a perturbation. Finally, it is worth to emphasize that modulation

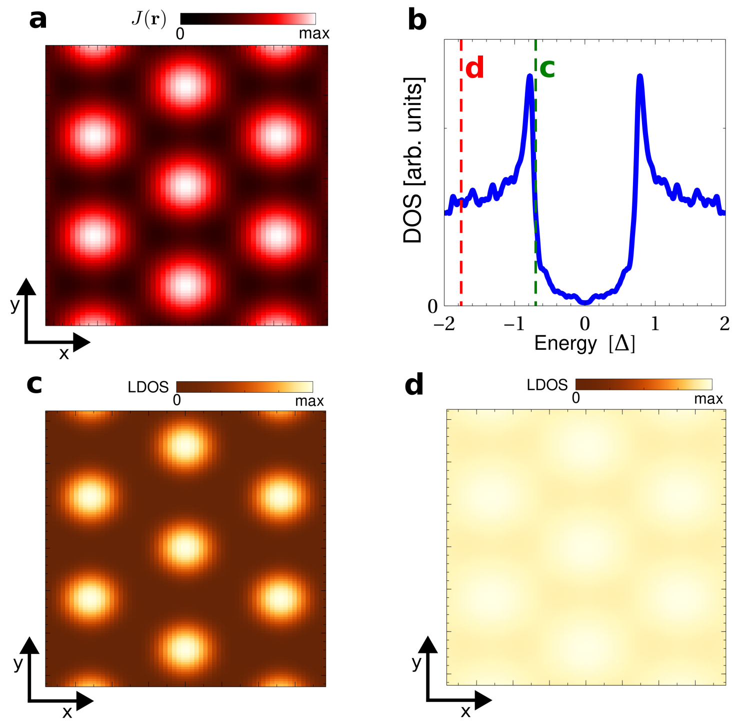

Figure S5: Exchange profile imposed in the supercell (a), and associated density of states including exchange, superconductivity and Rashba. The exchange profile is now define to have its minimum values at zero. Panel (c) shows the local density of states at the energy of the YSR bands, showing a strong localization where the exchange is stronger. For higher biases (panel d), the states are mostly delocalized in the supercell.

in other terms in the Hamiltonian can also be the driving force for localized YSR bands, yet we have found that modulation of the exchange is the most effective mechanism.

We finally comment on how the modulation of the Yu-Shiba-Rusinov bands can be rationalized. For this sake, let us enumerate the possible dependencies of the in-gap modes with the real-space location. First, let us consider the case in which there is a moiré pattern, but it has no influence on the electronic spectra. In this situation, it is clear that there would be no modulation in the YSR energies nor in the intensity, given that the induced exchange coupling and superconductivity will be effectively uniform. This is, of course, in stark contrast with our findings, so we can discard this trivial scenario. Second, let us assume that the moiré pattern gives rise to an exchange coupling modulated in space, but that each YSR state is decoupled from the neighbouring one, meaning that YSR bands do not appear. In this scenario, locally, we would have local exchange coupling with different strengths, which in turn gives rise to an in-gap YSR state whose energy is controlled by the local exchange coupling. This is the typical scenario for individual magnetic atoms in a superconducting surface, where the absorption site determines the effective exchange coupling leading to a site-dependent YSR state. In our system, we do not observe a YSR state whose energy is spatially dependent but rather a band of in-gap states with a spatially modulated intensity. Third, let us consider a case with a spatially modulated exchange field, and coupling between YSR states giving rise to a YSR band. In this situation, the whole bandwidth of the YSR can be observed at every location in the moiré pattern, yet with an intensity that depends on the specific location. This feature signals the emergence of a moiré YSR band and is analogous to the emergence of electronic moire states in other twisted van der Waals materials. Given the discussion above, we conclude that the scenario observed experimentally corresponds to the emergence of moiré YSR bands, leading to an electronic structure associated with the moiré length.

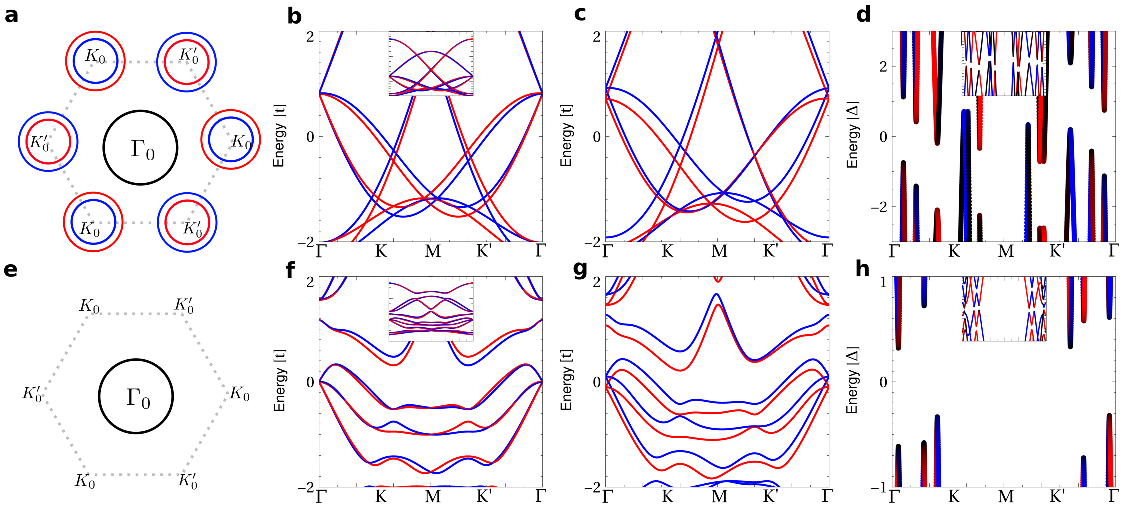

Figure S6: Band structure without (a-d) and with (e-h) charge density wave. Panel (a,e) show a sketch of the unfolded Fermi surface of $NbSe_2$ in the minimal unit cell. Panels (b,f) show the band structure with Ising SOC, panels (c,g) with Ising SOC and exchange, and panels (d,h) with Ising SOC, exchange, Rashba SOC and s-wave superconductivity. It is observed that in the absence of CDW the band structure with superconductivity is gapless (d). The band structures of (b,c,d,f,g,h) are computed in a 3×3 supercell.

Realistic tight-binding model for $CrBr_3/NbSe_2$ heterostructure

So far we have considered that the main contribution to the spin-orbit coupling comes from mirror symmetry breaking leading to a Rashba SOC term. However, dichalcogenides have an additional intrinsic term known as Ising spin-orbit coupling, that leads to a momentum-dependent splitting in the z-direction. Moreover, in the discussion so far we have included the effect of the modulated moiré potential, but the intrinsic charge density wave modulation was not considered. In this section, we show that the charge density wave order quenches the intrinsic effect of Ising spin-orbit coupling.

The charge density wave order is incorporated in the Hamiltonian as

$$H_{\text{CDW}} = \sum_{n,s} \epsilon_n c^\dagger_{n,s} c_{n,s} \tag{5}$$

where $\epsilon_n = \epsilon$ for bright sites and $\epsilon_n = 0$ for dark sites. Ising spin-orbit coupling is incorpo-

rated as a term of the form

$$H_{\text{Ising}} = i \sum_{\langle ij \rangle, s, s'} \gamma_{ij} \sigma_z^{s,s'} c_{i,s}^\dagger c_{j,s'} \tag{6}$$

where $\gamma_{ij} = \pm 1$ alternating signs in C_3 symmetric fashion.[S6]

To account for the existence of two pockets in the $NbSe_2$ band structure, we now use a modified tight binding model incorporating further neighbor hoppings, whose band structure at charge neutrality leads to a pocket around each valley and one around the Γ_0 point

$$H_{\text{kin}} = \sum_{i,j,s} t_{ij} c_{i,s}^\dagger c_{i,s} \tag{7}$$

with first neighbor hopping $0.1t$ and second neighbor hopping t, that reproduces the band dispersion and Fermi surface of $NbSe_2$.[S7] As a summary, the inclusion of Ising spin-orbit coupling in a minimal model without modulations is found to be dramatically detrimental to a topological superconducting state (Fig. S6). In particular, even modest values of this parameter turn a topological state into a gapless regime, a phenomenon that stems from the presence of a strong momentum-dependent spin splitting in the Fermi surface (Fig. S6a). To understand the underlying mechanism of this critical impact (Fig. S6(a-d)), and most importantly, how the CDW avoids this dramatic effect (Fig. S6(e-h)), it is convenient to see how the band structure is modified by this term. In the following, we will denote by Γ_0, K_0, K_0' the high symmetry points of the minimal unit cell, whereas Γ, K, K' will denote the high symmetry points of a 3×3 supercell.

For the sake of concreteness, in the following we will take a 3×3 supercell, which would correspond to the periodicity of the CDW order. In a minimal model without external modulations, the effect of Ising spin-orbit coupling is to create a momentum-dependent splitting in the band structure (Fig. S6a), keeping the $z-$direction of the spin a good quantum number. In particular, Ising spin-orbit coupling creates a positive exchange field around the K_0 pocket, an effective negative exchange field at the K_0' pocket, and an oscillating (and

nearly vanishing) exchange field around the Γ pocket (Fig. S6a). In particular, in the folded 3×3 band structure, this is seen as some states having a large spin-splitting, those coming from the K_0 and K_0' points, coexisting with states with a substantially smaller spin-splitting, coming from the Γ_0 pocket (Fig. S6b).

Upon introduction of the exchange bias created by $CrBr_3$, the combined effect of Ising spin-orbit coupling and exchange field breaks the degeneracy between states at $+k$ and states at $-k$ (Fig. S6c). This breaking of degeneracy is the ultimate responsible of the dramatically detrimental effect of Ising spin-orbit coupling. In particular, when a superconducting proximity effect is included, the absence of the degeneracy between states at $+k$ and $-k$ will drive the system to a gapless regime, as superconductivity pairs states with the same energy and opposite momentum (Fig. S6d). Note that such breaking of degeneracy does not happen for Rashba spin-orbit coupling in combination with an exchange field.

We now move on to consider the effect of the CDW order. The presence of a CDW creates scattering between the states in the Fermi surface, in particular, gapping away mostly the Fermi surface around K_0 and K_0' due to almost perfect nesting of those pockets with the wavevector of the CDW (Fig. S6e). In terms of the folded bandstructure, the states close to the Fermi surface now have a substantially smaller Ising splitting in the presence of the CDW (Fig. S6f). When an exchange bias is now included, the degeneracy breaking between states at $+k$ and states at $-k$ is now dramatically smaller in the presence of the CDW (Fig. S6g). As the degeneracy between those states is a necessary condition for having a gapped state, in the presence of the CDW, inclusion of superconductivity now leads to a fully gapped state (Fig. S6h).

Therefore, the presence of the charge density wave in the system has a crucial role for the emergence of a topological superconducting state, by quenching the effect of the critically detrimental effect of the Ising spin-orbit coupling. Given that upon inclusion of the CDW, the effective Fermi surface of the system is formed essentially by a pocket around the Γ_0 point, the combined effect of Ising spin-orbit coupling, charge density wave, and the different pockets

of $NbSe_2$, leads to an effective model that is captured by a triangular lattice at half-filling, as used in previous sections.

Spatially resolved YSR band energy and intensity

Fig. S7 covers a single grid spectroscopy measurement. To spatially resolve the YSR band energy and amplitude, the same procedure was applied to each spectrum. First, the spectrum was fitted by the two-band McMillan model with interband coupling. The fit excluded energy range of the YSR bands, i.e. region from −1 mV to 1 mV. Example of such a fit is shown in Fig. S7a, where the fitted curve is an average of all spectra of the grid spectroscopy. Next, the fit is subtracted from the spectrum, which results in two YSR bands as shown in Fig. S7b. Then an energy and dI/dV intensity of the YSR band at positive bias voltage was extracted by searching for maximum. Since this was done for each spectrum of the grid spectroscopy, we were able to obtain maps of energy of the YSR band and dI/dV intensity at the energy of the YSR band (Fig. S7d and Fig. S7e, respectively).

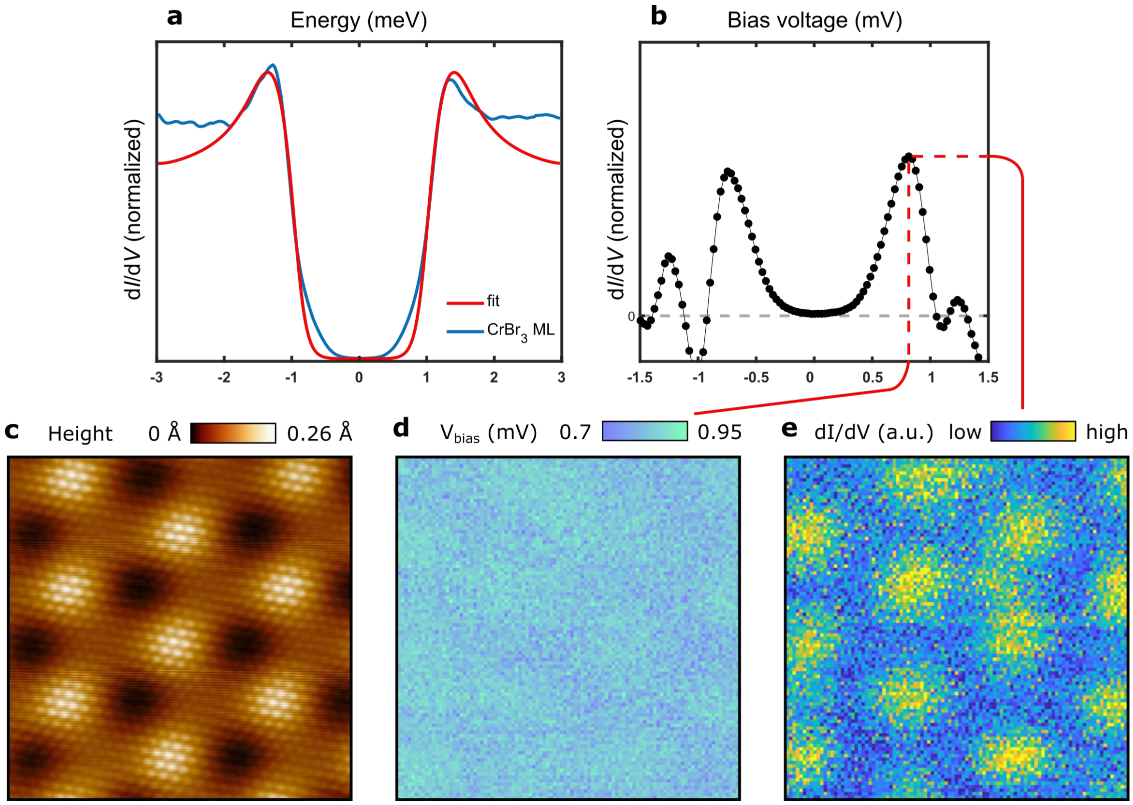

Figure S7: (a) Averaged dI/dV spectrum of the grid spectroscopy fitted by the two-band McMillan model with interband coupling. (b) YSR bands resulting from subtraction of the fit from the dI/dV spectrum. (c) 20×20 nm^2 STM image of an area where the grid spectroscopy was taken. (d,e) Spatially resolved YSR band energy (d) and intensity (e).

There is a very faint modulation of the extracted YSR band energy in space (Fig. S7d). This comes from the method of extracting this quantity from the measurement by searching for energy position of a maximum. However, no such energy modulation can be seen when looking at subtracted spectra across moiré sites (Fig. S8).

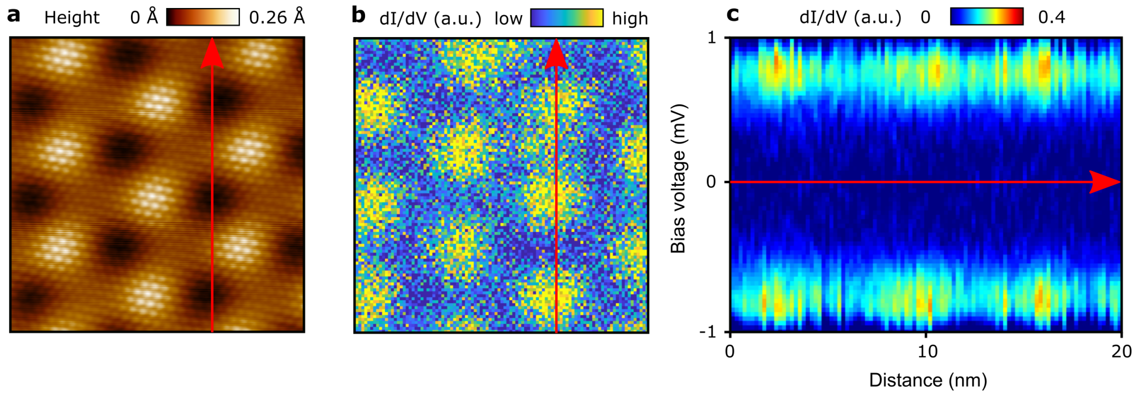

Figure S8: (a) STM image of $CrBr_3$ ML on $NbSe_2$ obtained at $V = 1.5$ V and $I = 300$ pA, image size is 20×20 nm^2. (b) dI/dV map at the energy of Shiba bands $V = 0.8$ mV. (c) dI/dV spectra along the red line displayed in (a) and (b).

Large bias dI/dV spectroscopy of $CrBr_3$ monolayer on $NbSe_2$

STM and STS reveal detailed information on how the interlayer atomic registry affects the local electronic structure of the $CrBr_3/NbSe_2$ van der Waals (vdW) heterostructure. To show the spatial modulation of the local electronic structure, we have recorded the dI/dV

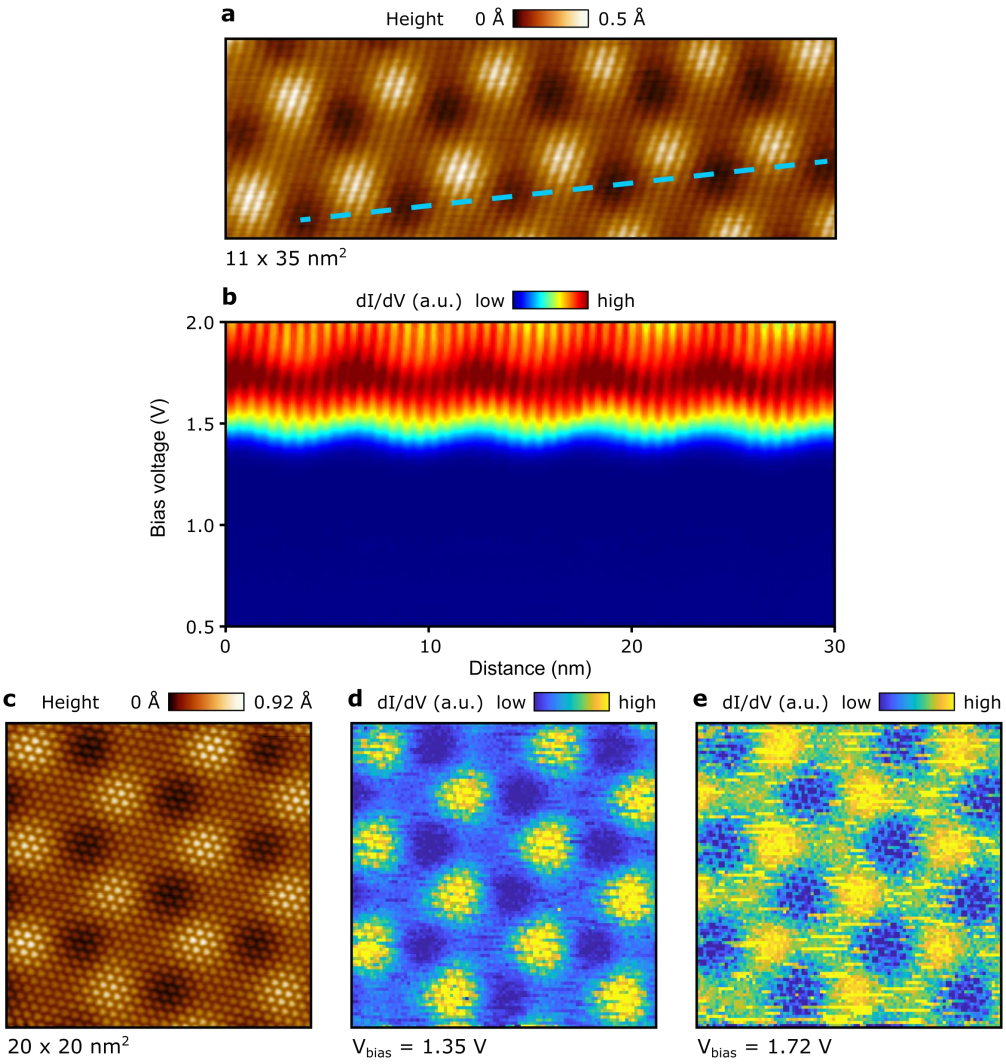

Figure S9: (b) dI/dV spectroscopy along the blue dashed line displayed on STM image shown in (a). The image size is 11×35 nm^2 and it is taken at $V_{\text{bias}} = 1.5$ V and $I = 200$ pA. (c) 20×20 nm^2 STM image taken at $V_{\text{bias}} = 1.5$ V and $I = 200$ pA. (d-e) dI/dV maps taken at an area shown in (c) and at corresponding V_{bias} values. All dI/dV spectra in this figure were normalized such that integral of the whole dI/dV spectrum is equal to one.

spectroscopy along the blue line indicated in Fig. S9a and the result is shown in Fig. S9b. The conduction band edge of $CrBr_3$ is modulated with exactly the periodicity of the moiré pattern of $CrBr_3/NbSe_2$ heterostructure, with magnitude of the modulation around 50 mV. This modulation with moiré pattern is also observed in the dI/dV maps (Fig. S9d,e) taken on the area shown in Fig. S9c. The periodic variation of local electronic structure as a consequence of the moiré pattern indicates the existence of a periodic electrostatic potential in this heterostructure.

References

(S1) Kezilebieke, S.; Silveira, O. J.; Huda, M. N.; Vaňo, V.; Aapro, M.; Ganguli, S. C.; Lahtinen, J.; Mansell, R.; van Dijken, S.; Foster, A. S.; Liljeroth, P. Electronic and Magnetic Characterization of Epitaxial $CrBr_3$ Monolayers on a Superconducting Substrate. *Adv. Mater.* **2021**, *33*, 2006850.

(S2) Lutchyn, R. M.; Sau, J. D.; Das Sarma, S. Majorana Fermions and a Topological Phase Transition in Semiconductor-Superconductor Heterostructures. *Phys. Rev. Lett.* **2010**, *105*, 077001.

(S3) Serlin, M.; Tschirhart, C. L.; Polshyn, H.; Zhang, Y.; Zhu, J.; Watanabe, K.; Taniguchi, T.; Balents, L.; Young, A. F. Intrinsic quantized anomalous Hall effect in a moiré heterostructure. *Science* **2020**, *367*, 900–903.

(S4) Sumiyoshi, H.; Fujimoto, S. Quantum Thermal Hall Effect in a Time-Reversal-Symmetry-Broken Topological Superconductor in Two Dimensions: Approach from Bulk Calculations. *J. Phys. Soc. Jpn.* **2013**, *82*, 023602.

(S5) Jiao, L.; Howard, S.; Ran, S.; Wang, Z.; Rodriguez, J. O.; Sigrist, M.; Wang, Z.; Butch, N. P.; Madhavan, V. Chiral superconductivity in heavy-fermion metal UTe_2. *Nature* **2020**, *579*, 523–527.

(S6) Kane, C. L.; Mele, E. J. Quantum Spin Hall Effect in Graphene. *Phys. Rev. Lett.* **2005**, *95*, 226801.

(S7) Fang, S.; Kuate Defo, R.; Shirodkar, S. N.; Lieu, S.; Tritsaris, G. A.; Kaxiras, E. Ab initio tight-binding Hamiltonian for transition metal dichalcogenides. *Phys. Rev. B* **2015**, *92*, 205108.
